# “Confidence comes with frequent practice”: health professionals’ perceptions of using manual vacuum aspiration after a training program

**DOI:** 10.1186/s12978-019-0683-z

**Published:** 2019-02-19

**Authors:** Maria Lisa Odland, Gladys Membe-Gadama, Ursula Kafulafula, Jon Øyvind Odland, Elisabeth Darj

**Affiliations:** 1 Department of Public Health and Nursing, Norwegian University of Science and Technology, Trondheim, Norway; 2 Queen Elizabeth Central Hospital, Blantyre, Malawi; 3 Kamuzu College of Nursing, Blantyre, Malawi; 4 College of Medicine, University of Malawi, Blantyre, Malawi; 5 University of Pretoria, Pretoria, South Africa; 6 Department of Obstetrics and Gynecology, St Olav’s Hospital, Trondheim, Norway; 7 Department of Women’s and Children’s Health, Uppsala University, Uppsala, Sweden

**Keywords:** Manual vacuum aspiration, MVA, Incomplete abortions, Post-abortion care, Health care providers

## Abstract

**Background:**

Malawi has one of the highest maternal mortality rates in the world, with unsafe abortion as a major contributor. Curettage is most frequently used as the surgical method for treating incomplete abortions, even though it is costly for an impoverished health system and the less expensive and safe manual vacuum aspiration (MVA) method is recommended.

**Methods:**

The aim of this 2016–17 study is to explore health worker’s perception of doing MVA 1 year after an educational intervention. Focus group discussions were recorded, transcribed verbatim, and analyzed using content analysis for interpreting the findings. A knowledge, attitude and practice survey was administered to health professionals to obtain background information before the MVA training program was introduced.

**Results:**

Prior to the training sessions, the participants demonstrated knowledge on abortion practices and had positive attitudes about participating in the service, but preferred curettage over MVA. The training was well received, and participants felt more confident in doing MVA after the intervention. However, focus group discussions revealed obstacles to perform MVA such as broken equipment and lack of support. Additionally, the training could have been more comprehensive. Still, the participants appreciated task-sharing and team work.

**Conclusion:**

Training sessions are considered useful in increasing the use of MVA. This study provides important insight on how to proceed in improving post-abortion care in a country where complications of unsafe abortion are common and the health system is low on resources.

## Plain English summary

The aim of this study was to explore health worker’s perception of using manual vacuum aspiration (MVA) 1 year after an educational intervention. The health personnel in Malawi who treat women with incomplete abortions are medical interns, clinical officers and nurses/midwives. The training was designed as a refresher course as health personnel usually are trained how to do MVA during their studies. Altogether, 53 health personnel received the training and filled out questionnaires on knowledge, attitude and practice before the training had started. After 1 year, focus group discussions were arranged with 33 of the previously trained health personnel. There was an almost equal number of nurses, clinical officers and medical interns in the survey and in the focus groups, which included fairly equal numbers of male and female participants and a good age spread among the participants. Even though the participants demonstrated knowledge on the benefits of using MVA prior to the training, most of the health personnel used curettage because of a lack of confidence, broken equipment and high gestational age of the expectant mothers. The training sessions were considered useful, but could have been extended to several days and have included actual patients. Our findings provide important insight on how to proceed in improving post-abortion care in a country where complications of unsafe abortion are common and the health system is low on resources.

## Background

Malawi is a small landlocked country in the southeastern part of Africa with a population of about 18 million [1]. It is a poor country with limited resources [2, 3]. Its maternal mortality ratio of 439 per 100,000 is one of the highest in the world [2–5]. A major contributor to this is unsafe abortion [6–8], and it has been estimated to be the cause of up to a quarter of the maternal mortalities in the country [9]. Unsafe abortion is defined by the World Health Organization (WHO) as “a procedure for termination of a pregnancy done by an individual who does not have the necessary training or in an environment not conforming to minimal medical standards*”,* and worldwide around 25 million unsafe abortions are performed every year [10]. It occurs most commonly in countries where there is a restrictive abortion law, like Malawi. Even though there are ongoing discussions about liberalizing the abortion law in this country, currently it is only legal to terminate a pregnancy in order to save a pregnant woman’s life [11, 12]. Nevertheless, there are about 140,000 induced abortions performed in Malawi every year [13]. Considering its restrictive abortion law many of these are unsafe and lead to complications that create a big burden for an already impoverished health system. A common issue involves retained products of conception that can lead to ongoing bleeding and infections that in the worst-case scenario can lead to hypovolemic shock and death [14]. Even though complications can happen after normal miscarriages or safe abortions, it is more likely to occur when pregnancy termination is conducted in an unsafe manner. In order to avoid further complications, incomplete abortions need to be treated with uterine evacuation. Medical treatment with misoprostol and vacuum aspiration is the preferred treatment in high-income countries, and their use is increasing in many parts of the developing world [15, 16]. However, in countries where misoprostol is accessible it has been used to induce abortions illegally [17]. A recent systematic review revealed several barriers to using misoprostol such as fear and apprehensions at policy, provider and community level, shortage of staffing and stock depletion [18]. These are factors that could contribute to most incomplete abortions in Malawi being treated surgically. Another concern is that patients can be lost to follow up and subsequently need attention because of serious septic abortions. Stock depletion of medicines is a common problem in Malawi [19], and thus it makes sense to treat patients surgically.

Surgical evacuation of the uterus can be done using traditional dilatation and curettage (D&C) or using vacuum aspiration [20]. According to the WHO vacuum aspiration is the recommended treatment in the first trimester, and can be achieved using electrical equipment or by using manual vacuum aspiration (MVA) [20–22]. Considering that MVA can be carried out without electricity and under local anesthetics, it is a cheaper and easier way to evacuate the uterus. It is also safer with less risks of complications such as uterine perforation, bleeding and Asherman’s Syndrome [20, 21]. Nurses/midwives are trained and allowed to evacuate the uterus with MVA in addition to clinical officers and doctors. It is the recommended surgical treatment in Malawi for incomplete abortions in the first trimester [23]. This option is also important in other low-income countries with huge patient loads and sparse resources. Unfortunately, studies have shown that the use of MVA is decreasing in Southern-Malawi and, concomitantly, the use of D&C is on the increase in many hospitals in spite of the mentioned guidelines [21, 23–25]. A follow-up study in the same area suggested that one reason for this is lack of trained personnel [26]. The latter constituted an incentive for initiating MVA-training of health personnel in a number of hospitals in Southern Malawi.

The use and implementation of MVA involves interplay between personal and contextual factors.

Our primary aim of the present study was to explore Malawian health workers’ perceptions about the use of MVA in post-abortion care (PAC) at three public hospitals 1 year after an educational intervention.

## Materials and methods

### Study design

We used a knowledge, attitude and practice (KAP) survey, and a qualitative part which involved focus group discussions (FGDs) among health professionals. The survey follows the WHO questionnaires on KAP [27], although our questions were adjusted to focus on post-abortion care issues in Malawi. The questionnaire was pretested on eight individuals from different professions including nurses, gynecologists, general practitioners and other professions before conducting the survey and no changes were required. The KAP survey provided an assessment of the participants’ knowledge, attitudes and practice prior to an intervention. The FGDs gave the PAC providers an opportunity to interact and discuss commonalities and differences in their methods of treatment. A semi-structured topic guide was used to conduct the FGDs which was based on a review of the literature and discussions among the authors. It probed their perceptions on carrying out MVAs including obstacles and enablers, and personal feelings about being comfortable conducting MVAs and the need for additional training. At the end of the session, additional thoughts and discussion on the implementation of MVA were encouraged.

### Study setting

The selection of the study sites was opportunistic as all three hospitals had previously been shown to have a low use of MVA [24, 25]. The southern region of Malawi is the most populated region and features a high unmet need of family planning (20%) [5]. At least half of the complications after abortions are treated in public facilities [28], and the central hospital included in this study treats around 200 patients with incomplete abortions every month [24, 25]. A high number of incomplete abortions are also treated in the district hospitals. Therefore, one tertiary central hospital and two smaller district hospitals in the southern region of Malawi were included. The MVA-training program was initiated in April 2016, and constitutes an intervention study. The training was only provided in the selected hospitals and not in other hospitals in the country.

### Study participants

The intervention targeted all pertinent health personnel allowed to treat patients with incomplete abortions and included registered nurses, clinical officers, and recently graduated doctors (medical interns). Clinical officers have a separate and shorter training program than doctors, but do have many of the same responsibilities such as anesthesia, diagnosis and treatment. They are an important cadre, especially in the district hospitals where there are few doctors. A meta-analysis has shown that they are capable of carrying out physician’s procedures such as caesarean sections [29, 30]. The training program was offered to nurses and clinical officers at the selected district hospitals and medical interns at the referral hospital. The training was planned for 12 participants per session and enrolment was voluntary. One training session was conducted at each hospital in April 2016, and one extra session was added at the referral hospital to accommodate a new rotation of interns later that year. All of the participants completed the KAP questionnaire *prior to* receiving their training, and were invited to participate in FGDs 1 year after the intervention. None declined, but some participants were missing in the follow up interviews due to rotations to different departments/facilities or they were unable to leave duty at the given time period for the interviews.

### The training intervention

The training was organized as a refresher course as basic knowledge about MVA should have been obtained within their respective curricula in medical or nursing school. It included basic theory and practice for conducting MVA using the ten-step guidelines recommended by Ipas [31], as well as bimanual examination in order to determine uterine size, estimating the gestational age, choosing the right size of the MVA cannula, antiseptic preparations and the procedure itself. Information on sterilizing equipment was given. The procedure was carried out on pelvic models that were specially made for MVA-training [32]. There was no formal practical examination for the participants, but all had to demonstrate the whole MVA procedure using the pelvic models in the presence of the course providers. The training course was delivered by a local gynecology consultant with previous teaching experience and was supervised by the principal investigator – both of whom are physicians experienced in treating incomplete abortions. Nurses experienced in doing MVA also assisted in the training sessions.

### Data collection

#### Knowledge, attitude and practice

Before the training session commenced, all of the participants completed a questionnaire on KAP to assess their knowledge, attitudes and practices of PAC and MVA. It also sought information on providers sociodemographic information, background knowledge on abortions and incomplete abortions in Malawi, attitudes about treating patients with incomplete abortions and previous experience conducting uterine evacuation of any kind.

#### Focus group discussions

One year after the training sessions, six focus group discussions were conducted among the participants. At the district hospitals, separate groups were organized for clinical officers and nurses to enable them to discuss freely and thereby avoiding any influence related to social norms and hierarchy. Since English is an official language in Malawi and the main language in the work setting, it was used for the FGDs and thereby circumventing the need of a translator. All the FGDs took place in non-public hospital meeting rooms and had a duration of 45–60 min. At the outset of a session the participants were informed of its intent and that they could withdraw at any time without giving a reason. They were told to share as much information as they felt comfortable with. Only their age, gender and professions were recorded, but not their names.

The first and last author of the current paper served as moderators, and alternated in taking notes. With the permission of the participants, the FGD discussions were audio recorded.

### Data analysis

The background information from the KAP survey was tabulated and considered as descriptive data. The feedback from the FGDs was analyzed using qualitative content analysis as outlined by Graneheim and Lundman [33]. The interviews were transcribed verbatim by the first author. The first step in the analysis was to distill meaningful units from these transcriptions, which were then condensed and coded by the first author. All authors read the transcripts, discussed how to interpret the data, and suggested subcategories, which were then formalized and re-reviewed to reach final consensus agreement. This was done to capture the manifested meanings. In addition, selected statements by the participants and pertinent quotes were selected to illustrate important perceptions about MVA and related clinical practices.

## Results

### Knowledge, attitude and practice

Summaries of KAP questionnaire answers are presented in Tables 1, 2 and 3. All health personnel (100%) were aware of symptoms, and the recommended treatment for incomplete abortions (Table 1). However, only a third knew what the actual Malawian abortion law was, and two thirds believed that abortion was not legal under any circumstance. A positive attitude regarding treatment of incomplete abortions as an important part of the job was stated by 96.2% of the participants, while 56.6% of the participants preferred MVA to D&C and regarded it as easier to use and safer (Table 2). However, only 17% of them used MVA the last time they performed uterine evacuation (Table 3).

**Table 1:** Background knowledge about abortion care among the participants in the Malawian study *prior to* receiving their MVA training (*n* = 53)

Question	Knowledge *n (%)*
*Is abortion legal?*	
Yes	0 (0)
No	33 (62.3)
Only to save a pregnant woman’s life?	19 (35.8)
Not answered	1 (1.9)
*Is abortion common in Malawi?*	
Yes	51 (96.2)
No	1 (1.9)
Not answered	1 (1.9)
*Symptoms of incomplete abortion* ^a^	
Vaginal Bleeding following amenorrhea	53 (100)
Lower abdominal Pain	41 (77.4)
Backache	5 (9.4)
*Complications of incomplete abortion* ^a^	
Sepsis/infection	35 (66.0)
Anemia	18 (40.0)
Death	13 (24.5)
Hypovolemic Shock	12 (22.6)
Hemorrhage	10 (18.9)
Asherman Syndrome/Infertility	7 (13.3)
*Recommended surgical treatment for incomplete abortions*	
Manual Vacuum Aspiration	53 (100)
Curettage	0 (0)

^a^Several possible answers

**Table 2:** Study participants’ attitudes about incomplete abortions *prior to* their MVA training (*n* = 53)

Issue	Attitude *n (%)*
*Treating women with incomplete abortions is an important part of my job* ^a^	
Strongly agree	42 (79.2)
Agree	9 (17.0)
Disagree	1 (1.9)
Strongly disagree	0 (0)
Not answered	1 (1.9)
*I wish it was not part of my job?*	
Strongly disagree	34 (66.7)
Disagree	9 (17.6)
Agree	6 (11.8)
Strongly agree	2 (3.9)
*I prefer not to treat these women because abortion is a sin*	
No	52 (98.1)
Yes	1 (1.9)
*Which method do I prefer for uterine evacuation and why?*	
Manual Vacuum Aspiration	30 (56.6)
Easier	14 (46.7)
Safer	12 (40.0)
The one taught	3 (10.0)
Other^a^	1 (3.7)
Curettage	22 (41.5)
The one taught	9 (40.9)
Other^a^	5 (22.8)
Easier	5 (22.7)
Safer	3 (13.6)
Medical/Cytotec	1 (1.9)
The one taught	1 (100)

^a^Other reasons given included: being cost-effective, less time consuming or being more confident

**Table 3:** Study participants’ practice with *MVA prior* to the training sessions (*n* = 53)

Question	Practice *n (%)*
*How many times have you performed uterine evacuation?*	
0	8 (15.1)
1	3 (5.7)
>3	39 (73.6)
Not answered	3 (5.6)
*Which method did you use the last time and why?*	
Curettage	32 (60.4)
The method I prefer	4 (12.9)
The method available	7 (22.6)
The one I was told	4 (12.9)
Other^a^	16 (51.6)
Manual Vacuum Aspiration	9 (17.0)
The method I prefer	6 (40.0)
The method available	0 (0.0)
The one I was told	6 (40.0)
Other^a^	3 (20.0)
Medical/Cytotec	0 (0.0)
Not answered	12 (22.6)
*Is time a limiting factor when choosing treatment?*	
Yes	29 (55.8)
No	23 (44.2)
*Is lack of equipment a problem?*	
Yes	46 (86.8)
No	7 (13.2)

^a^Other factors given included safer, 2nd trimester, easier, and most familiar method

### Focus group discussions

There was an even distribution of nurses, clinical officers and medical interns who attended the FGDs, as well as of gender and age (Table 4). The key issues that emerged from them about training and MVA practices are summarized in Table 5.

**Table 4:** Identity of the participants in the follow-up FGD sessions (*n* = 33)

Hospital	Clinical Officers	Nurses	Interns	Age	Male	Female
District Hospital 1	5	6	0	24–48	7	4
District Hospital 2	4	6	0	28–40	4	6
Central Hospital	0	0	12	20–30	7	5
Total	9	12	12	20–48	18	15

**Table 5:** Overview of the main categories and subcategories emerging from the follow-up FDGs with Malawian health care providers

Categories	Subcategories
*Previous experience*	Learned it in school
	Learned by practice
	Training session
*Obstacles encountered in conducting MVAs*	Lack of equipment
	Lack of time
	Lack of encouragement
*Task-shifting*	Positive that nurses can do MVA
	Nurses liking the responsibility
	Better teamwork
*Motivation for doing MVA*	It is better for the patient
	Following guidelines

### Previous experience

It is clear that the health personnel had different experiences in the past with MVA, and this seems to be reflected in their views of the training refresher program. Some participants had learned how to conduct MVA during their studies and had considerable practical experience with it. Nevertheless, they were not feeling confident about it. Several nurses had not performed any MVAs since finishing nursing school, while a few were very experienced.

*“It was like a refresher. I did not have any experience of how to do a MVA, but now I have been reminded of how to do it.” (*Nurse 1, 2nd District Hospital).

Not all health personnel had received extensive training in the past. On the other hand, they had gained working experience. As mentioned the health system in Malawi is low on resources and personnel, and therefore it is common to “see one, do one and teach one”. The common path for them was to learn MVA procedures from senior staff and colleagues, who thus served as role models. This had influenced their MVA treatment and performance skills. In this context, it was beneficial to review MVA theory and procedures.

*“It seems the interns were doing more evacs than MVA back then, so we weren’t really exposed to a lot of MVAs as students*.*”* (Intern 8, 1st Session Central Hospital).

Feedback indicated that the training sessions provided were appreciated. It was evident that they were reminded of skills they had forgotten, and thereby became more confident. Hence, the refresher training was regarded as helpful, and had provided them with the additional knowledge and confidence needed to perform MVA procedures.

*“We increased our knowledge with the training. We became more confident in doing the procedure.”* (Clinical Officer 3, 1st District Hospital).

### Obstacles encountered in conducting MVAs

Even though the FDG participants described the training as useful and important, they mentioned several obstacles they encounter in their work setting such as a lack of properly working MVA kits and of other equipment such as protective gear.

*“Sometimes we don’t have sterile gloves and speculums. And sometimes we don’t have water and electricity. Then it’s difficult to do MVAs.”* (Nurse 3, 2nd District Hospital).

One opinion was that MVA is a time-consuming procedure. Even though they were aware that this procedure was safer and better for the patients, their overall workload and lack of staff made them continue using curettage.

*“I think MVA is simple, but it requires patience.”* (Nurse 3, 2nd District Hospital).

Doctors and clinical officers are called to operating theaters when everything is prepared for them, including general anesthesia, and their involvement in doing a D&C is of short duration. However, an MVA requires the administration of pain relief, and time to take effect, and the cannula needs to be prepared. Moreover, the equipment requirement was mentioned as another reason that rendered MVA a time-consuming process.

*“I think it’s good to have training, but the MVAs we have do not have enough suction so it takes longer for one to do MVA than curettage, and with the number of patients one has waiting you prefer to do curettage. Not because you are not comfortable doing an MVA, but because it takes longer than curettage.”* (Intern 4, 1st FGD Central Hospital).

Lack of encouragement from the hospital leaders also influenced what treatment method was being used. It became evident that they do not care what treatment was chosen as long as the patient was treated and discharged. Support from the leadership would also facilitate the provision of the proper equipment and guidance about what treatment method is recommended.

*“We cannot say there is any specific support from the management. We only report. The policy is there, they encourage us, but maybe the implementation down here is different. Before the training was done, it was long time since we had any training. So, even though the policy is there, the capacity is not.”* (Nurse 1, 2nd District Hospital).

*“Confidence comes with frequent practice. We had a debate among a number of nurses. We are trained to do MVAs, but they were not done. The clinical officers had to push until we got a room to do MVAs. Then we got more training and top up with a number of nurses. However, they have not got enough practice and no confidence.”* (Clinical Officer 1, 1st District Hospital).

### Task-shifting

There was agreement that MVA was a safer and cheaper method of treating incomplete abortions, even though obstacles to doing the procedure existed. Another aspect that proved to be valuable was the idea of task shifting. Since nurses can do MVA, but not curettage, it might reduce the heavy workload of clinical officers and doctors.

*“For me it has improved because the nurses can do some of our workload. We can divide the work between us”.* (Clinical Officer 2, 1st District Hospital).

Clearly it is of value that clinical officers get more time for other critical patients. It is also better for patients with incomplete abortions to be treated forthwith so that they do not have to wait for the clinical officers to be available. Nurses felt that this was a positive option and enjoyed the extra responsibility.

*“I think the training was important. In the past, abortions were only treated by clinicians who are not available in the wards all the time. While nurses are always in the ward”.* (Nurse 2, 1st District Hospital)

It was also an impression that the health personnel were now working more as a team. It was not just an issue of task-shifting, but also task-sharing. They were now able to work as a team to ensure patients were getting the most appropriate treatment and when they needed it.

*“We cannot say it is shifting from clinical officers to nurses. It’s more about collaboration.”* (Clinical Officer 3, 1st District Hospital)

Unfortunately, collaboration was not evident in all the hospitals. In the referral hospital, evacuations were described more as an intern’s tasks, and the nurses could not perform any MVAs even though they had the skills.

*“They are registered to do so, but it is more about that it is the intern’s job to do it, so I haven’t come across a nurse who has offered to do it. They have the knowledge, but they leave it to us.”* (Intern 4, 2nd FGD Central Hospital).

One intern in the same group had a different take on the situation. Lack of time and personnel could be reasons why these nurses did not do MVAs and help the interns with their heavy workload. Also, the ratio of patients per nurse is very high at the central hospitals, and most likely higher than patients per doctor in the gynecological ward.

*… it might be wrong to say they have a lot of time, because sometimes they have staff shortages [too].* (Intern 1, 2nd FGD Central Hospital).

### Motivation for doing MVA

Motivation seems to be an important factor for doing MVA. Since the health care providers were working in busy hospitals with a big patient load, they were not motivated to perform a procedure that is more time consuming than others. On the other hand, participants knew that MVA is safer and should be done according to the guidelines. MVA can also be done as an outpatient procedure, which means patients do not have to wait so long before the procedure nor stay overnight.

*“It’s much better to do MVA. Normally when we do evacuations there is more risk of complications. When you do MVA you don’t have the same risk of complications.”* (Clinical Officer 2, 2nd District Hospital)

The nurses were motivated and content about having this extra task which could improve the situation for the patients and help the clinical officers.

*“I feel MVAs will help improve the situation because these MVA can be done without clinicians. It helps many patients and a clinician doesn’t have to be around. We really have a very experienced team here to take care of the patients. We are ready to take care of them.”* (Nurse 5, 1st District Hospital)

*“MVA is safer and nurses can also do it which saves your time.”* (Clinical Officer 2, 2nd District Hospital)

## Discussion

Our study shows that conducting a refresher training among health personnel was well received by the participants. The health workers are well aware of the high prevalence of abortions and the possible complications. They also have knowledge of MVA as the recommended and preferred treatment, but the majority use curettage due to obstacles at different levels. The participants felt more confident after the training, and were content that they now had more treatment options. An additional benefit was more teamwork and task-sharing at the district hospitals, where the nurses can care for many of the patients with incomplete abortions. The extensive shortage of healthcare workers made the WHO develop global recommendations and guidelines on task-sharing as a tool for countries to implement safe task-sharing processes [34]. Training with task-shifting has already been proven successful in other developing countries, regarding processes like cesarean sections and hernia repair [35, 36]. Our analysis of the material made us see other factors than just the training which influenced the practice; direct factors, like lack of equipment, number of staff and current policies, and indirect factors, like team-work and leadership [37].

The individual health worker’s experience and previous education, practice, support, access to supervision and acceptance of MVA influences the confidence of using the suggested tool, irrespective of their appreciation of the training given. The KAP survey revealed that health personnel think that treating patients with incomplete abortions is an important part of their job. They did not oppose to treating these patients because of personal moral beliefs in contrast to a study from Zimbabwe where the health personnel referred to the women in negative terms [38]. The health workers in our study did not regard such patients as immoral, and were dedicated to providing them with the best possible treatment. Lack of time, lack of experience and a high patient load were seen as obstacles similar to another study [39]. The nurses were content to have a task they can perform on their own, and enjoyed the extra responsibility of sharing the treatment of incomplete abortions with clinical officers as also seen in a previous qualitative study from Uganda [39].

Moreover, the clinical officers in the district hospitals were pleased that the nurses could share their workload. An anticipation is that if senior doctors and clinical officers encourage the nurses their confidence in performing MVAs will evolve. Lack of encouragement from colleagues and the leadership was seen as an obstacle and should be addressed. It is clear that the motivation is there and small measures could be taken to try increase their confidence. Encouraging health workers to follow guidelines would be instrumental to increase use of MVA. Our study demonstrates that the health workers were knowledgeable about the guidelines and recommendation to use MVA. However, this was not built on by the leadership nor was MVA use implemented. In our assessment, this constitutes a missed opportunity to improve PAC.

A previous Malawian study has shown that training and task-shifting efforts seem to have had a positive effect on maternal mortality [40]. Furthermore, experiences with task-shifting in other developing countries such as India and Sierra Leone yielded comparable results when surgery was performed by less educated health personnel [34–36]. Another study has also shown that the outcomes are comparable when incomplete abortions are diagnosed and treated by midwives instead of physicians [41].

Most specialists work at the larger hospitals, and have the opportunity to engage and ensure that their patients are being treated according to the existing guidelines. Concurrently, specialists have an overarching responsibility to the rural District Hospitals in supervising clinical activities there. However, the lack of consultants in gynecology makes it very hard to adequately supervise all such facilities. Nevertheless, it is essential for all countries to insert a continuum of care, and this is especially so in low-income regions with high maternal mortality.

The guidelines by the WHO, International Federation of Gynecology and Obstetrics (FIGO) and the Malawi Ministry of Health clearly state that vacuum aspiration is the preferred surgical method for treatment of incomplete abortions in the first trimester and that the use of curettage should be decreased to the minimum [21, 23, 42]. Our experience is that training can be done repeatedly and effectively with little cost. One or two experienced individuals in a hospital can be enough to conduct regular training sessions for their co-workers, while lack of equipment needs to be solved at the administrative level. A previous study from Malawi has shown that MVA is 29% cheaper than performing curettage [43] in addition to being safer and having fewer complications [20]. Its formal adoption would help to reduce maternal mortality and the costs of treating serious complications after abortions within health systems with limited resources [20, 21, 43].

### Trustworthiness

The collaboration between Norwegian and Malawian researchers, obstetricians, gynecologists and midwives with experiences of treating patients with incomplete abortions was important for gaining an in-depth understanding of the data and to ensure credibility. The different personal and professional backgrounds of the authors were also helpful in the indicated classification/illustration/interpretation exercises. The first author lived in Malawi for several years and was onsite during the entire data collection process to ensure consistency, dependability and thereby building trust. During the whole process, she kept in mind how her role as a medical doctor, and a white expatriate woman, could have affected the data collection. To enhance the transferability of the project full details on the setting, the participants, data collection and qualitative analyses procedures are provided.

### Strengths and limitations

A strength of our study is that the follow-up FGDs were employed to explore the views on MVA and PAC of local health personnel. It supplemented the KAP survey data and gave us insight not only about the participants’ knowledge on the Malawian abortion law, symptoms and complications after abortions, but also their attitudes about abortions and the methods and practices in use at a community/hospital level. Training sessions are common in Malawi and other developing countries, but there is little data on follow ups. A possible limitation is whether the participants were sharing their actual perceptions rather than what they thought the researchers wanted to hear, or what was expected of them. However, the participants were speaking in a freely and uninhibited manner, and were encouraged by the moderator to be honest and give critical feedback.

## Conclusions

Our study shows that the organized MVA-training provided was well received the health personnel and had a role in increasing the use of MVA in the treatment of incomplete abortions. A lack of properly working equipment, insufficient staff and time, and an absence of leadership support were major obstacles to employing MVA. Furthermore, our study suggests that attitudes and change in practices to use another and recommended uterine evacuation method is complex as it challenges various concerns, such as the individual health provider’s perspective, team work, leadership and policy issues. Change in behavior therefore needs to be addressed at several levels, and a health systems approach is recommended.

## Abbreviations

D&C: Dilatation and Curettage
FIGO: International Federation of Gynecology and Obstetrics
MVA: Manual Vacuum Aspiration
PAC: post-abortion care
